# A systematic scoping review on the consequences of stress-related hyperglycaemia

**DOI:** 10.1371/journal.pone.0194952

**Published:** 2018-04-06

**Authors:** Elena Olariu, Nicholas Pooley, Aurélie Danel, Montserrat Miret, Jean-Charles Preiser

**Affiliations:** 1 PHMR Ltd, London, United Kingdom; 2 Nestlé Health Science, Vevey, Switzerland; 3 Department of Intensive Care, Erasme University Hospital, Université Libre de Bruxelles, Brussels, Belgium; Azienda Ospedaliero Universitaria Careggi, ITALY

## Abstract

**Background:**

Stress-related hyperglycaemia (SHG) is commonly seen in acutely ill patients and has been associated with poor outcomes in many studies performed in different acute care settings. We aimed to review the available evidence describing the associations between SHG and different outcomes in acutely ill patients admitted to an ICU. Study designs, populations, and outcome measures used in observational studies were analysed.

**Methods:**

We conducted a systematic scoping review of observational studies following the Joanna Briggs methodology. Medline, Embase, and the Cochrane Library were searched for publications between January 2000 and December 2015 that reported on SHG and mortality, infection rate, length of stay, time on ventilation, blood transfusions, renal replacement therapy, or acquired weakness.

**Results:**

The search yielded 3,063 articles, of which 43 articles were included (totalling 536,476 patients). Overall, the identified studies were heterogeneous in study conduct, SHG definition, blood glucose measurements and monitoring, treatment protocol, and outcome reporting. The most frequently reported outcomes were mortality (38 studies), ICU and hospital length of stay (23 and 18 studies, respectively), and duration of mechanical ventilation (13 studies). The majority of these studies (40 studies) compared the reported outcomes in patients who experienced SHG with those who did not. Fourteen studies (35.9%) identified an association between hyperglycaemia and increased mortality (odds ratios ranging from 1.13 to 2.76). Five studies identified hyperglycaemia as an independent risk factor for increased infection rates, and one identified it as an independent predictor of increased ICU length of stay.

**Discussion:**

SHG was consistently associated with poor outcomes. However, the wide divergences in the literature mandate standardisation of measuring and monitoring SHG and the creation of a consensus on SHG definition. A better comparability between practices will improve our knowledge on SHG consequences and management.

## Introduction

Hyperglycaemia is frequently observed in critically ill patients [1] and can occur in the absence of pre-existing glucose intolerance or diabetes mellitus. In critical illness, hyperglycaemia appears to be the result of stress, hence its denomination of stress-related hyperglycaemia (SHG). However, there is currently no universal threshold for the definition of SHG [2], yielding very different estimates of its prevalence (from 19.9% when blood glucose (BG) levels were higher than 153 mg/dL (8.5 mmol/L) [3] to 75% when the threshold was 110 mg/dL (6.1 mmol/L) [4]).

Regardless of its prevalence, several studies have shown that SHG is associated with complications, prolonged stay in intensive care units (ICUs) and hospitals, increased incidence of infection, increased mortality, and increased use of resources [5–9]. In survivors of critical illness, an association between SHG during hospitalisation and subsequent diabetes has been shown in several studies [10–14], with patients with SHG having an increased risk of incident diabetes [13].

However, the optimal management of SHG is still unknown, as prospective interventional studies targeting predefined BG levels with insulin yielded highly conflictual results. In a single-centre interventional trial conducted in 2001, Van den Berghe and colleagues showed the benefits of treating SHG with intensive insulin therapy and its direct clinical implications in ICU patients. Reduced morbidity (bloodstream infections, acute renal failure requiring dialysis, red-cell transfusions, ICU-acquired weakness) and mortality were observed in surgical ICU patients whose target BG levels were 80–110 mg/dL (4.4–6.1 mmol/L) [4].Subsequently, other interventional trials were unable to reproduce the results of the pioneering trial [15–21]; for example, the NICE-SUGAR study found that a target BG level of <180 mg/dL was associated with a reduced 90-day mortality rate compared with 81–108 mg/dL [22]. Similarly, the Glucontrol study did not demonstrate a difference in mortality between patients randomised to a target BG range of 79.2–111.6 mg/dL and 140.4–180 mg/dL [23]. Therefore, there is a lack of external validation of the target BG levels observed in previous trials and no widely accepted SHG definition. This is both a result of heterogeneous patient characteristics and management and divergence in individual study design, including BG target, type of BG measurement, outcome variable (e.g. 28-, 90-, or 180-day mortality, or ICU or hospital mortality), setting, and available resources [24]. The published systematic reviews and meta-analyses pooled highly heterogeneous data [24–26].

Hence, a clearer view is urgently needed (1) to better characterize a clinically relevant and widely acceptable definition of SHG and (2) to allow better identification of the type of patients and situations in which SHG is associated with a poor outcome, and in whom therapeutic strategies of SHG should be assessed. This may require a flexible approach in which SHG is not absolutely defined for all patients, but can be adapted according to the type of patient and their circumstances. It should also be noted that SHG is not the same as high blood glucose levels, which may resolve without the need for treatment. Better SHG definitions will allow patients requiring SHG treatment to be more clearly identified. This systematic scoping review aimed to provide a basis for these more targeted research questions from observational data of hyperglycaemia in the acutely ill patient.

## Methods

The Joanna Briggs Institute guidelines on conducting systematic scoping reviews were followed [27–29]. This methodology summarizes the evidence available on a topic in order to convey the breadth and depth of that topic.

### Research question

The research question for this review was: ‘What are the characteristics, breadth, and results of the existing research conducted in observational settings on the clinical burden of hyperglycaemia in acutely ill adult patients admitted to ICUs?’

### Information sources and search strategy

A search strategy combining both MeSH and free-text terms for hyperglycaemia and ICU settings was developed to retrieve articles of interest in the following databases: Medline; Medline In-Process Citations & Daily Update; Embase; and the Cochrane Library. The search strategy was designed in Medline and Medline In-Process and then translated to the other databases (S1 Table).

Searches were limited to English language studies and the period between January 2000 and December 2015. Additionally, publications were excluded electronically if they were indexed as case reports, case series, editorials, or letters. In addition to the electronic searches, the 2014 and 2015 proceedings of nine conferences were screened (S2 Table).

### Eligibility criteria

Studies were included irrespective of the definition of SHG, if they were observational and reported data in adult patients (≥18 years) in mixed and trauma ICUs on hyperglycaemia and either of the following outcomes: mortality, infections, hospital/ICU length of stay, time on ventilation, ICU-acquired weakness, blood transfusions, and renal replacement therapy. Reviews, systematic reviews, and studies with fewer than ten hyperglycaemic patients were excluded, as were studies that compared the performance of different insulin protocols.

### Study selection process

Titles and abstracts were screened by three reviewers against the agreed inclusion and exclusion criteria. Disagreements between reviewers were resolved by consensus and the reasons for exclusion were recorded only at the full-text stage.

### Charting the data

The research team developed a data extraction tool that included the following items:

- article identifiers (authors, year of publication, objective)
- study identifiers (sample size, design, country, length of follow-up, inclusion and exclusion criteria)
- setting and population (age, gender, co-morbidities, reason for admission, Acute Physiology and Chronic Health Evaluation [APACHE] severity scores)
- method of BG measurement, insulin protocols, hyperglycaemia treatment protocols, definition of hyperglycaemia
- outcome measures: mortality, infections, ICU and hospital length of stay, time on mechanical ventilation, blood transfusions, renal replacement therapy, and ICU-acquired weakness.
- Data were extracted by one team member and verified by a second reviewer.

### Collating, summarising, and reporting the results

A descriptive numerical summary of the characteristics of the included studies was performed. Tables and graphs were created to reflect the overall number of studies included, study designs and settings, publication years, the characteristics of the study populations, the outcomes reported, and the countries where the studies were conducted. In line with scoping reviews’ methodology, an assessment of the quality of the included studies was not performed.

## Results

### Studies’ characteristics

**Fig 1:**
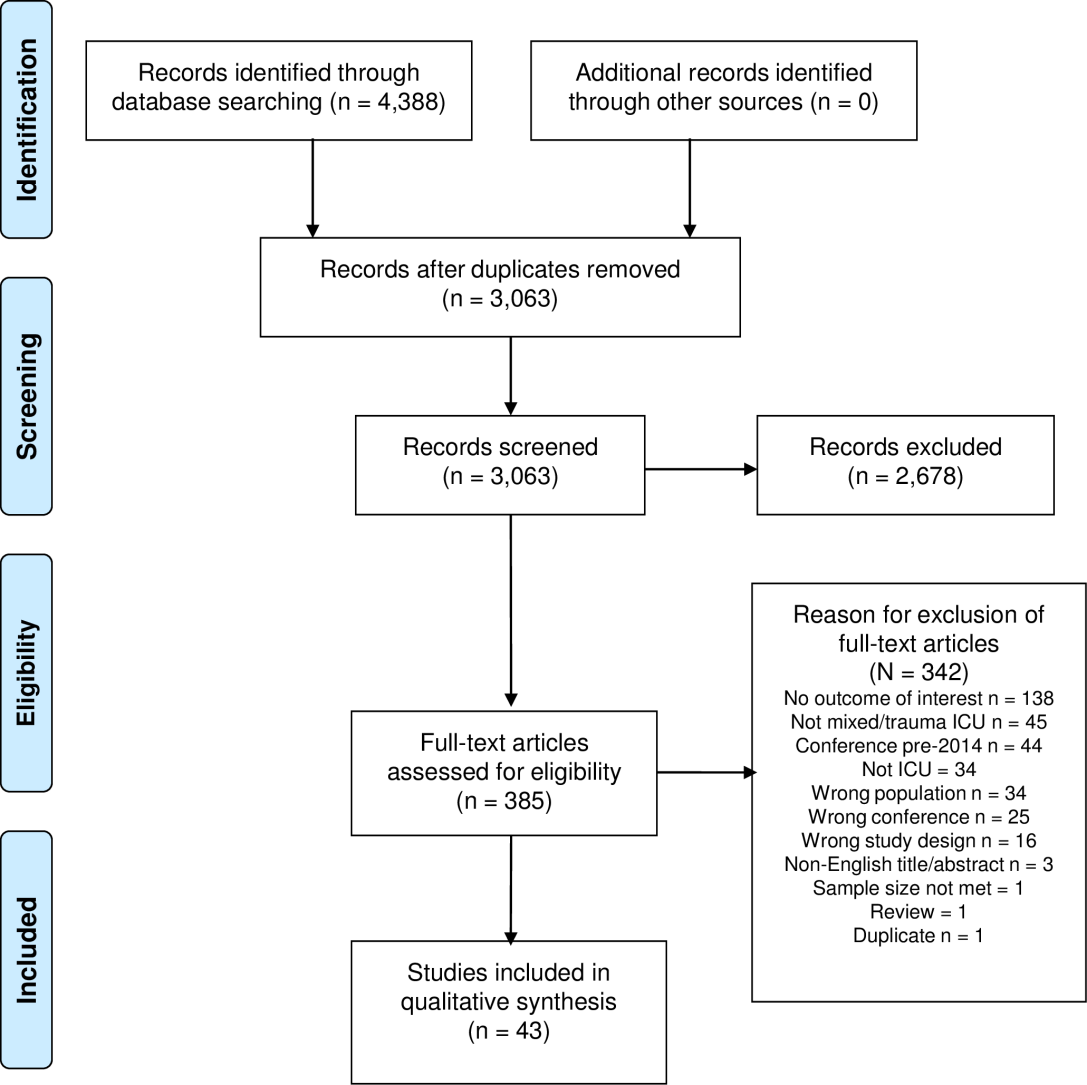
Flow of studies in the systematic scoping review and reasons for exclusion.

A total of 3,063 articles were retrieved. After title and abstract screening, 385 records were kept for full-text retrieval and 43 articles were included at full-text review (Fig 1). The results presented here are for 42 studies (536,476 patients), as two articles [30,31] were linked and reported data from the same study.

#### Setting

The reported data were mostly collected in the USA (Table 1), the commonest ICU type was trauma; however, there were more patients included in studies from mixed ICU. Trauma ICUs comprised general trauma and more specialist centres; aside from general ICUs, the most frequently reported centres cared for patients with head/brain/neurological injury, while one study reported data from a burn and trauma unit and another described patients with orthopaedic trauma (Table 1).

**Table 1:** Setting: Study location and ICU type.

Setting	No. of studies	No. of patients	References
Study location			
USA and Canada	28	523,271	[30,32–58]
Europe	6	9,560	[3,59–63]
Middle East	3	1,152	[64–66]
Asia	2	328	[67,68]
South America	2	1,165	[69,70]
Australia	1	1,000	[71]
ICU type			
Trauma	17	8,383	[33,34,36–39,41,42,44,45,47,49,53,55,59,66,67]
Mixed medical/surgical	8	255,544	[3,32,51,54,56,61,62,69]
Mixed medical/surgery/cardiac/coronary	5	267,655	[35,40,50,58,70]
Head/brain/neurologic trauma	5	1,822	[48,60,64,65,68]
Medical	2	500	[30,63]
General	2	1,170	[46,71]
Burn and trauma	1	609	[43]
Orthopaedic trauma	1	187	[57]
Mixed neurologic medical/surgical/trauma	1	606	[52]

#### Blood glucose

Several BG thresholds were used to define SHG, ranging from 100 mg/dL (5.6 mmol/L) to 300 mg/dL (16.7 mmol/L). The SHG classification with the highest number of patients was >150 mg/dL (201,608), followed by >180 mg/dL (198,465), and >200 mg/dL (40,354) (S1 Fig). In several studies, patients were classified into different groups depending on the magnitude of HG.

Clinical practice was also highly variable and very often incompletely reported. The sampling site was capillary, arterial, or venous. The meter used was either a blood gas analyser, a point-of-care glucometer, or a central laboratory interface (S1 Fig). Ten studies used more than one of these meters to assess BG levels.

There was considerable heterogeneity and occasional ambiguity in the reporting of the timing of samples used to assess BG levels. While some studies reported the timescale of samples used (e.g. those taken in the first 24 or 48 h), others reported the frequency at which samples were obtained (e.g. hourly or daily). In many studies, BG was measured both at admission and during ICU stay, with most measurements obtained within the first 72 h after admission (S1 Fig). BG was usually calculated as an average of all measures taken or as the highest value recorded in a given time period. It was only very rarely reported as a time-weighted average [54].

When reported, the target BG ranges were also very heterogeneous (S1 Fig); while six studies reported the use of a treatment protocol for hyperglycaemia [3,44,49,58,61,62], others reported that control of hyperglycaemia was not formalised and at the discretion of the attending critical care physician [46,60]. The most common target range was 80–110 mg/dL, which was used in 4 studies. The ranges used in the remaining studies varied, but most targeted BG levels of <150 mg/dL (S1 Fig).

#### Patients’ characteristics

Besides demographic data (age, gender), the descriptions of clinical characteristics of the patients were variable, including the type of admission, and severity score (S2 Fig). The majority of studies reported the number of patients with diabetes in their population; however, the subsequent processes were varied, with some studies including a mix of diabetic and non-diabetic patients while others excluded diabetic patients entirely. More studies were conducted in mixed populations of both diabetic and non-diabetic patients than in separate populations (S2 Table).

### Study outcomes

**Fig 2:**
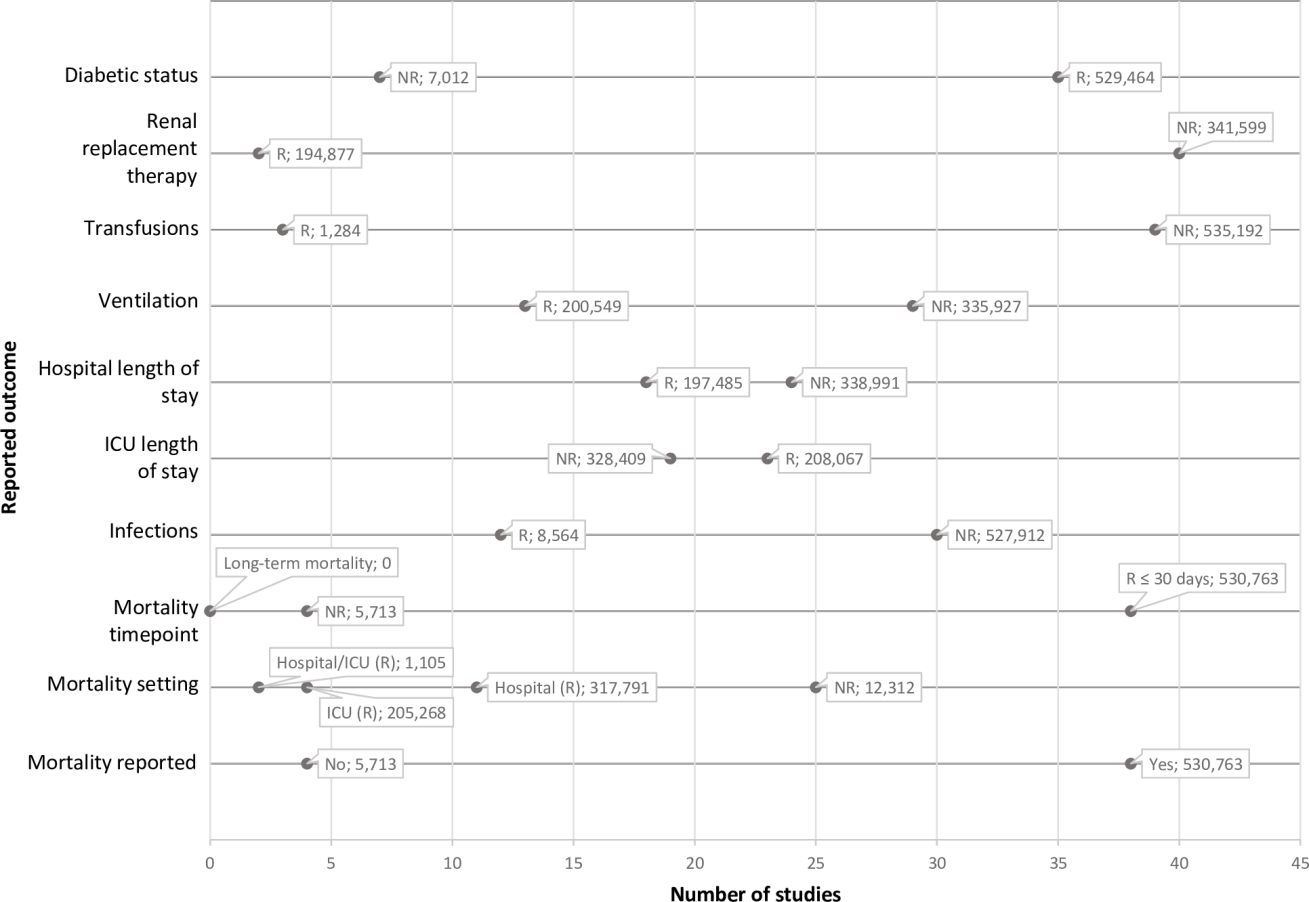
Distribution of study characteristics and outcomes in observational studies conducted in trauma and mixed ICUs. Callouts might overlap for label categories with the same number of studies. ICU, intensive care unit; NR, not reported; R, reported.

Most of the included studies assessed the impact of high BG levels on clinical outcomes as the primary study objective. However, the outcome variables reported varied widely (Fig 2). Although part of the scoping review, ICU-acquired weakness was not reported as an outcome in any of the included studies.

#### SHG and mortality

Mortality was reported in 38 studies across both trauma and mixed ICUs (Fig 2; S2 Fig). Most of the studies reported hospital mortality, or short-term mortality (7-, 14-, 21-, or 30-day mortality), and only one reported ICU mortality (Fig 2). No studies reported data on long-term mortality rates. The ranges of hospital mortality differed widely, ranging between 3.1–43.0% [34,35,40,42,46,50,52,55,56,58,59]; reported ICU mortality ranged between 1.2–35.6% [3,32,54,60,62,63,69]. The heterogeneity in the types of mortality reported precluded the calculation of a mean mortality rate from the included studies. Of note, there was no report of the observed/expected mortality rate.

S2 Table shows unadjusted mortality levels in hyperglycaemic and non-hyperglycaemic patients in trauma and mixed ICUs. Where reported, ORs ranged between 1.00–17.1. However, mortality varied across all studies in terms of the cut-off point for BG, diabetes status, ICU type, underlying disease, type of insulin control, and mortality measurement time-point.

Mortality levels were stratified by diabetic status in two studies [56,58]; one of them showed that patients with diabetes and mean BG between 110 mg/dL (6.1 mmol/L)– 180 mg/dL (10.0 mmol/L) had a lower mortality rate than patients with diabetes and mean BG between 80 mg/dL (4.4 mmol/L)– 110 mg/dL (6.1 mmol/L) [56]. Two studies reported mortality levels exclusively in diabetic patients [30,50].

Fourteen studies that assessed the impact of hyperglycaemia on mortality also determined whether hyperglycaemia was an independent risk factor for mortality [3,32,34,37,40,42,47–50,52,54,56,64]. Most of the studies showed that higher BG levels were associated with a higher risk of mortality even after adjusting for confounding variables, where reported ORs ranged between 1.00–17.1.

#### Infections in hyperglycaemic and non-hyperglycaemic patients

Twelve publications reported data on infections in ICUs (Fig 2 and S2 Table) from a total of 8,564 patients. The percentages of patients with infections varied from a low 12.5% (BG >150 mg/dL [8.3 mmol/L]) [41] to a high 61% (BG 140–219 mg/dL [7.8–12.2 mmol/L]) [42] (S2 Table). Details on the type of infections were provided in all studies: they included bloodstream, respiratory, genito-urinary, and surgical site infections.

Eight studies assessed whether hyperglycaemia was a risk factor for developing infections [34,36–39,42,57,62], and five of these [34,36,38,39,57] identified it as an independent risk factor, where reported, ORs ranged between 0.44–5.02. Across these latter studies, hyperglycaemia as an independent risk factor was expressed as a hyperglycaemic index [57] (ORa = 1.8, 95% CI 1.3–2.5), as BG levels ≥200 mg/dL (11.1 mmol/L) (P = 0.02, no odds ratios reported) [36], (P = 0.007, no odds ratios reported) [38] or >135 mg/dL (7.5 mmol/L) [34] (urinary tract infections: ORa = 3.3, 95% CI 1.21–8.8; pneumonia: ORa = 2.8, 95% CI 0.98–8.0) or as a pattern of glucose control [39].

#### Hyperglycaemia and length of stay in ICU/hospital

ICU and hospital stays were reported by many studies, although not all studies reported both variables. Length of stay was consistently reported across studies and settings as measures of the central tendency (mean or median), and was usually stratified by BG levels. Among studies that reported ICU length of stay (Fig 2), the mean duration was 10.9 days (range 1.9–34) in patients with SHG; this value includes patients categorised as having moderate or severe SHG. In patients with normal BG levels, the mean length of ICU stay was 9.1 days (range 1–30 days). It should be noted that some studies excluded patients who stayed in the ICU for less than 24 or 48 h, which may have affected the findings. The role of hyperglycaemia as a predictor of ICU length of stay was investigated in only one trauma ICU study [34].

#### Resource use in hyperglycaemic and non-hyperglycaemic patients

More studies reported time on mechanical ventilation (13 studies; 200,549 patients) (Fig 2) than blood transfusions (three studies; 1,284 patients) or renal replacement therapy (two studies; 194,877 patients). Time on ventilation was longer (range: 1 day [41,45]– 25 days [55]) for patients with hyperglycaemia than for those without, and this difference was found to be significant in five studies [37,39,41,42,61] (S2 Table). Hyperglycaemic patients were administered more units of blood (3.7 units SD = 2.5) than non-hyperglycaemic patients (3.1 units SD = 2.3) on average, but this difference was not statistically significant [38].

Two studies reported data on renal replacement treatment [54,63], revealing a higher number of hyperglycaemic patients undergoing dialysis than non-hyperglycaemic patients (4.2% versus 2.4%) [54] (S2 Table).

None of the included studies assessed whether hyperglycaemia was a risk factor for increased time on mechanical ventilation, an increased number of blood transfusions, or renal replacement therapy.

## Discussion

This systematic scoping review was performed to identify the characteristics, extent, and results of existing research conducted in observational settings on the association between occurrence of hyperglycaemia in adult patients admitted in ICUs and various outcomes. To the authors’ knowledge, this is the first scoping review to systematically assess the clinical burden of SHG in ICUs in observational studies, as the majority of evidence synthesis data currently available on hyperglycaemia have focused mainly on RCTs, which can differ from the exact conditions of clinical practice [72]. Even though an association between SHG and worsened outcomes was acknowledged by 43% of the studies identified (as reported by 18 of the 42 included studies reporting on either mortality, infections or ICU length of stay), our results revealed great variability in terms of reporting and conduct of the included studies, illustrating a high heterogeneity in clinical practice across settings, patients, and geographies.

Among the sources of heterogeneity, the types of ICUs widely differed, especially considering the lack of standardization in the definitions of trauma, mixed, medical, surgical units. Fewer studies reported data on mixed than on trauma ICU patients, although more patients were admitted in mixed than in trauma ICUs. Few studies provided detailed information on how BG was measured (frequency or time-point of measures, site of blood sampling), or the techniques used to monitor or analyse BG in critically ill patients, in spite of the current recommendation to report these data [73]. No studies conducted on trauma ICU patients reported such evidence, while differences in the accuracy of measurement can be relevant, especially in case of peripheral hypoperfusion, or in the presence of physico-chemical confounding factors [73]. This information is essential to accurately compare and understand the results of the various studies. Regarding outcomes reporting, very few studies had a defined time-point to measure mortality, for example. Additionally, hypoglycaemia was reported in only nine studies [3,30,51,52,54,55,61,63,70], in spite of the current recommendations that it should be reported alongside hyperglycaemia due to its association with increased mortality and morbidity [73]. This lack of important information could reflect a lower focus interest for dysglycemia in ICU patients than in patients with diabetes, in relation with the much higher complexity of the critically ill.

In spite of these broad disparities, the SHG is associated with a significant clinical burden defined *a priori* as a combination of patients’ severity of disease, including the outcome variables improved during the pioneering study [4] and the use of available resources. Hopefully, the SHG-related clinical burden could be decreased by the appropriate control, prevention, treatment, and monitoring, when specific categories of patients and situations could be identified. Unfortunately, the current evidence is probably too heterogeneous to allow such identification. In fact, the results of this study are comparable to those reported in 2008 by Eslami et al. [74] in a systematic review on quality indicators for tight glycaemic control in critically ill patients. The same review also identified high variability and ambiguity in the definitions and threshold values for reporting hyperglycaemia, noting the reduced comparability among studies for these reasons [74]. In line with other systematic reviews [13,25,75,76], our review has also pointed out that the heterogeneous nature of the methods used in studies in this field prevents meta-analysis of data, making narrative summaries more appropriate. However, narrative summaries are not as informative as meta-analyses for clinical decision-making processes as they do not allow the calculation of pooled effect estimates [77]. Moreover, the issue of whether HG should be considered as a marker of the severity of disease or as a potentially modifiable risk factor cannot be solved with the current set of available data.

Future studies should focus more on providing details on BG sampling techniques, BG measurement protocols, and on clearly defining outcomes, including their time-points of measurement. Furthermore, studies should more frequently report on the potential differences in the clinical burden of SHG between diabetic and non-diabetic patients, Indeed, the optimal BG value could differ between diabetic or chronically hyperglycaemic patients [78].

Other unexplored outcomes of clinical burden, such as ICU-acquired weakness or nursing workload, the amount of transfusion should also become the focus of future observational studies. Of particular interest is ICU-acquired weakness as its impact goes beyond the hospitalisation phase; it specifically contributes to the physical limitations in ICU survivors that are associated with reduced health-related quality of life and higher one-year mortality [79].

Our scoping review has several limitations. First, our searches were limited to studies published in English, potentially leading to language bias and exclusion of relevant articles published in other languages. Second, ICU-acquired weakness was defined according to the most recent terminology, excluding terms such as polyneuromyopathy, critical illness myopathy, or polyneuropathy. This was done at both the screening and data-extraction stage, which may explain why no studies were found to report data on ICU-acquired weakness. Finally, scoping reviews are not intended to assess the quality of the literature analysed. Thus, the conclusions of this review are based on the existence of studies rather their intrinsic quality. Nevertheless, this scoping review provides a comprehensive overview of the existing research on hyperglycaemia in ICUs in observational settings, by reporting data collected from more than 500,000 critically ill patients, which is one of the highest numbers of patients ever studied in intensive care medicine.

## Conclusions

The clinical consequences of SHG represent adverse outcomes for acutely ill patients. The understanding of the magnitude of this burden is limited due to the great variability observed in studies’ reporting and conduct. This highlights an urgent need for a consensus and unified criteria for measuring and controlling SHG, if better care is to be provided. Recommendations have been previously published by clinical experts in the acute care field [80], making these recommendations clinically meaningful. These recommendations include standardisation of blood glucose sampling, as well as the metrics to report glycaemic control. Such recommendations were published with the aim to improve glycaemic control in daily clinical practice, while also minimising the disparities facilitating the interpretation and comparison of clinical trials. Individualised thresholds for different patient subgroups might be the way forward in the management of SHG, but this approach will only become the standard in clinical practice if an improvement in patient outcomes is supported by a consistent and homogeneously conducted and reported body of evidence.

## Supporting information

S1 Fig Blood glucose (BG) variables by number of studies and patients. A. Stress hyperglycaemia (SHG) definition. B. BG sampling site. C. Meter used for BG sampling. POC/gluc, point-of-care/glucometer; BGA, blood gas analyser; Lab, laboratory D. Timing of BG sampling. E. Target BG range. NR, not reported.
*Data from the study by Falciglia et al. (2009) were not included in this graph, as the study compiled findings from 173 hospitals with more than 250,000 patients. As it also considered multiple HG definition categories (all >111 mg/dL), inclusion of this study would have skewed the findings.
(PPTX)

S2 Fig Patient characteristics by number of studies and patients. A. Acute Physiology and Chronic Health Evaluation (APACHE) score. B. Mortality type. ICU, intensive care unit; NR, not reported.
(PPTX)

S1 Table Search strategy for Medline and Medline In-Process. (DOCX)

S2 Table Mortality, infections, length of stay, and resource use in patients with or without hyperglycaemia in trauma and mixed ICUs. ^a^Respiratory, genito-urinary, bloodstream; ^b^respiratory infections; ^c^genitourinary tract infections; ^d^blood infections; ^e^intra-abdominal infections; ^f^skin/soft tissue infections; ^g^respiratory infections, genito-urinary tract infections, wound or skin infections; ^h^pneumonia, urinary tract infections, bacteraemia, intra-abdominal abscess, wound infection, open fracture infection; ^i^pneumonia, line sepsis, bacteraemia, wound infection, abscess; ^j^overall ICU mortality; ^k^week 1 mortality; ^l^week 2 mortality; ^m^week 3 mortality; ^n^bacteraemia; ^o^urinary tract infections; ^p^pneumonia in the third week; ^q^surgical site infections; ^r^pneumonia, urinary tract infection, bloodstream infection, surgical site infection, intra-abdominal abscess, *Clostridium difficile* colitis, meningitis, and sinusitis; ^s^skin/wound infections; ^t^other infections; ^u^pneumonia; ^v^wound infections.
BG, blood glucose; CI, confidence interval; CIT, continuous intravenous regular human insulin infusion; HG, hyperglycaemia; HGI, hyperglycaemic index; HR, hazard ratio; ICU = intensive care unit; IIT, intensive insulin control; IQR = interquartile range; ISS = injury severity score; max, maximum; NA = not applicable; NPH = neutral protamine Hagedorn; NR, not reported; OR, odds ratio; ORa, odds ratio adjusted; PH, persistent hyperglycaemia; RR, risk ratio; RRa, risk ratio adjusted; SD, standard deviation; SE, standard error; SEM, standard error of the mean; SIT, supplemental intermittent intravenous regular human insulin therapy; TBI, traumatic brain injury; TIR-hi, time in targeted blood glucose range above the median value; TIR-lo, time in targeted blood glucose range below the median value. *P<0.05; **P<0.01; †Nosocomial infections.
(DOC)
