# Supplementary material for: A systematic scoping review on the consequences of stress-related hyperglycaemia
Source: PLoS One. 2018 Apr 6;13(4):e0194952. doi: 10.1371/journal.pone.0194952 (PMC5889160; doi:10.1371/journal.pone.0194952)
Supplement: S1 Fig — A. Stress hyperglycaemia (SHG) definition. B. BG sampling site. C. Meter used for BG sampling. POC/gluc, point-of-care/glucometer; BGA, blood gas analyser; Lab, laboratory D. Timing of BG sampling. E. Target BG range. NR, not reported. *Data from the study by Falciglia et al. (2009) were not included in this graph, as the study compiled findings from 173 hospitals with more than 250,000 patients. As it also considered multiple HG definition categories (all >111 mg/dL), inclusion of this study would have skewed the findings. (PPTX) [file pone.0194952.s001.pptx]

## Slide 1
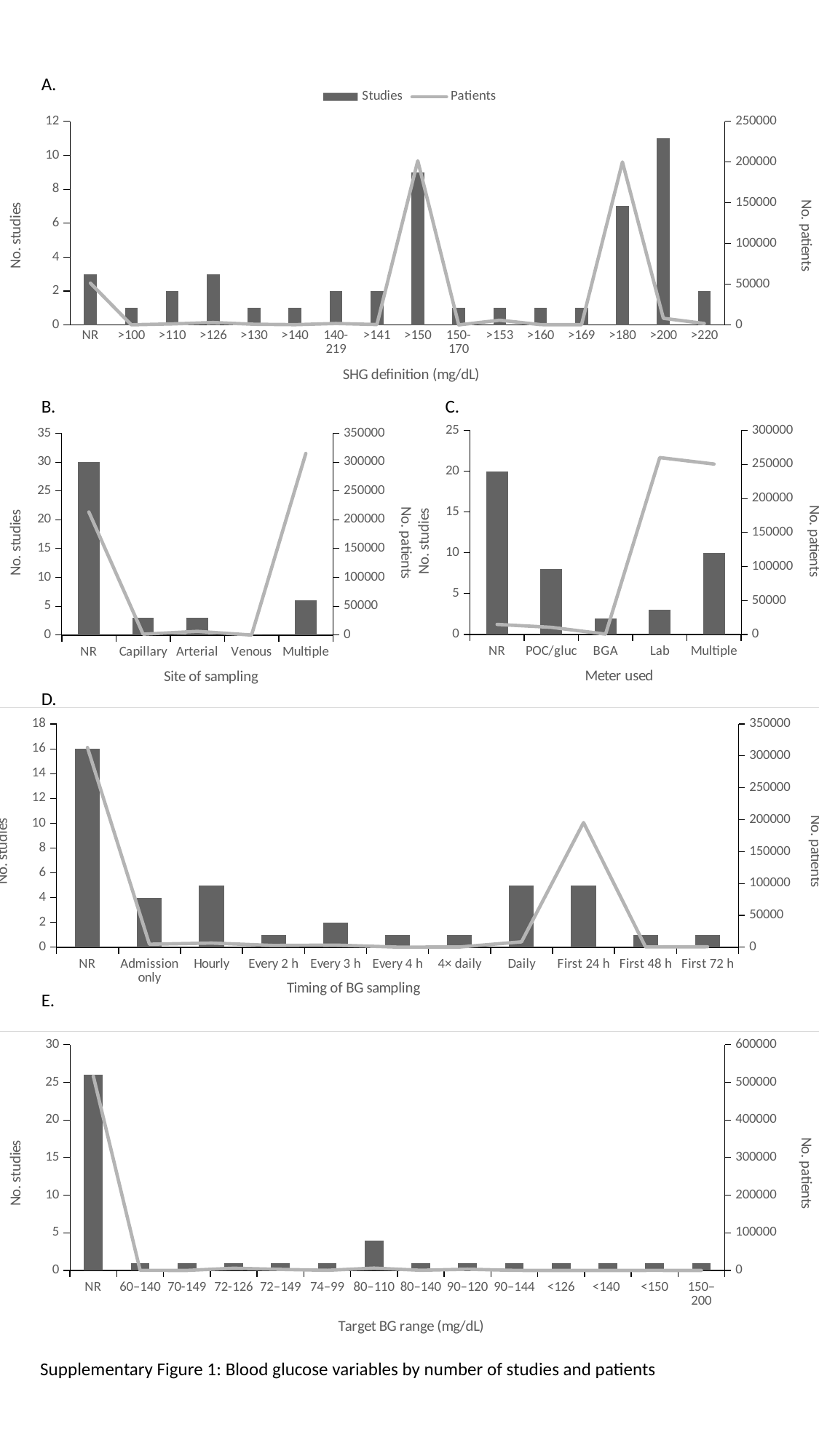

A.
### Chart
| Category | | |
|---|---|---|
| NR | 3.0 | 51378.0 |
| >100 | 1.0 | 208.0 |
| >110 | 2.0 | 1295.0 |
| >126 | 3.0 | 2991.0 |
| >130 | 1.0 | 862.0 |
| >140 | 1.0 | 338.0 |
| 140-219 | 2.0 | 1838.0 |
| >141 | 2.0 | 498.0 |
| >150 | 9.0 | 201608.0 |
| 150-170 | 1.0 | 66.0 |
| >153 | 1.0 | 5828.0 |
| >160 | 1.0 | 386.0 |
| >169 | 1.0 | 279.0 |
| >180 | 7.0 | 200185.0 |
| >200 | 11.0 | 8211.0 |
| >220 | 2.0 | 1838.0 |B.
C.
### Chart
| Category | | |
|---|---|---|
| NR | 20.0 | 14779.0 |
| POC/gluc | 8.0 | 10529.0 |
| BGA | 2.0 | 566.0 |
| Lab | 3.0 | 260044.0 |
| Multiple | 10.0 | 250558.0 |
### Chart
| Category | | |
|---|---|---|
| NR | 30.0 | 213526.0 |
| Capillary | 3.0 | 1490.0 |
| Arterial | 3.0 | 6394.0 |
| Venous | 0.0 | 0.0 |
| Multiple | 6.0 | 315066.0 |D.
### Chart
| Category | | |
|---|---|---|
| NR | 16.0 | 313070.0 |
| Admission only | 4.0 | 4733.0 |
| Hourly | 5.0 | 6622.0 |
| Every 2 h | 1.0 | 2782.0 |
| Every 3 h | 2.0 | 3402.0 |
| Every 4 h | 1.0 | 228.0 |
| 4× daily | 1.0 | 386.0 |
| Daily | 5.0 | 8480.0 |
| First 24 h | 5.0 | 195478.0 |
| First 48 h | 1.0 | 516.0 |
| First 72 h | 1.0 | 779.0 |E.
### Chart
| Category | | |
|---|---|---|
| NR | 26.0 | 516588.0 |
| 60–140 | 1.0 | 338.0 |
| 70-149 | 1.0 | 66.0 |
| 72-126 | 1.0 | 5828.0 |
| 72–149 | 1.0 | 2782.0 |
| 74–99 | 1.0 | 279.0 |
| 80–110 | 4.0 | 6267.0 |
| 80–140 | 1.0 | 395.0 |
| 90–120 | 1.0 | 3297.0 |
| 90–144 | 1.0 | 228.0 |
| <126 | 1.0 | 105.0 |
| <140 | 1.0 | 103.0 |
| <150 | 1.0 | 170.0 |
| 150–200 | 1.0 | 30.0 |Supplementary Figure 1: Blood glucose variables by number of studies and patients
