# Supplementary material for: A systematic scoping review on the consequences of stress-related hyperglycaemia
Source: PLoS One. 2018 Apr 6;13(4):e0194952. doi: 10.1371/journal.pone.0194952 (PMC5889160; doi:10.1371/journal.pone.0194952)
Supplement: S2 Fig — A. Acute Physiology and Chronic Health Evaluation (APACHE) score. B. Mortality type. ICU, intensive care unit; NR, not reported. (PPTX) [file pone.0194952.s002.pptx]

## Slide 1
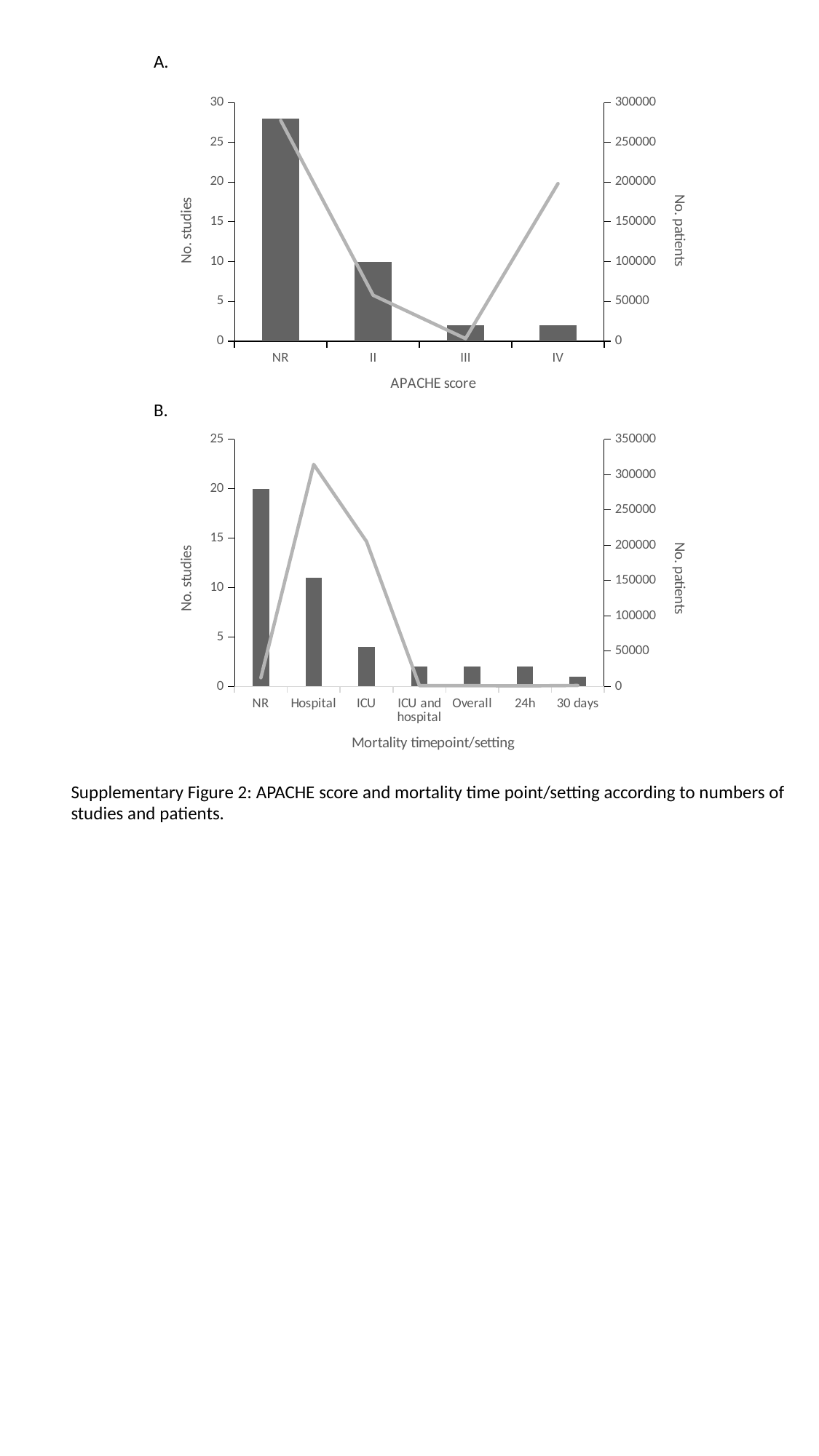

A.
### Chart
| Category | | |
|---|---|---|
| NR | 28.0 | 277297.0 |
| II | 10.0 | 57797.0 |
| III | 2.0 | 3313.0 |
| IV | 2.0 | 198069.0 |B.
### Chart
| Category | | |
|---|---|---|
| NR | 20.0 | 12312.0 |
| Hospital | 11.0 | 314595.0 |
| ICU | 4.0 | 205268.0 |
| ICU and hospital | 2.0 | 1105.0 |
| Overall | 2.0 | 1090.0 |
| 24h | 2.0 | 772.0 |
| 30 days | 1.0 | 1334.0 |Supplementary Figure 2: APACHE score and mortality time point/setting according to numbers of studies and patients.
