# Supplementary material for: A systematic scoping review on the consequences of stress-related hyperglycaemia
Source: PLoS One. 2018 Apr 6;13(4):e0194952. doi: 10.1371/journal.pone.0194952 (PMC5889160; doi:10.1371/journal.pone.0194952)
Supplement: S1 Table — (DOCX) [file pone.0194952.s003.docx]

**Supplementary table 1. Search strategy for Medline and Medline In-Process**

| **Search date** | **Limits** | **Database** | **Search strategy** | **Conference proceedings searched** |
| --- | --- | --- | --- | --- |
| Searched 19/01/16 via OvidSP interface | Limited to 2000 onwards, English language, human studies.  Excludes editorials, letters, case reports | Ovid MEDLINE(R) In-Process & Other Non-Indexed Citations and Ovid MEDLINE(R) <1946 to Present> | 1 exp Hyperglycemia/ (28561)  2 hyperglyc$.ti,ab. (46437)  3 ((high$ or height$ or excess$ or elevate$ or raise$) adj2 (blood adj1 (glucose or sugar))).ti,ab. (3663)  4 or/1-3 (62781)  5 exp Intensive Care Units/ (61434)  6 ((intensive or special) adj1 (care adj1 (unit$ or ward$))).ti,ab. (78001)  7 icu.ti,ab. (34769)  8 critical care/ or intensive care/ (42025)  9 (critical$ adj2 (care or ill$)).ti,ab. (51199)  10 or/5-9 (167305)  11 4 and 10 (2179)  12 11 not (animals/ not (human/ and animals/)) (2126)  13 limit 12 to (english language and yr="2000 -Current") (1800)  14 13 not (editorial or letter or case reports).pt. (1630)  1630 results. | 1. the American Diabetes Association (ADA) 2. the European Association for the Study of Diabetes (EASD) 3. Advanced Technologies and Treatments for Diabetes (ATTD) 4. the Diabetes Technology Society (DTS) 5. the European Society of Intensive Care Medicine (ESICM) 6. the International Symposium on Intensive Care & Emergency Medicine (ISICEM) 7. the Society of Critical Care Medicine (SCCM) 8. the American Society for Parenteral and Enteral Nutrition (ASPEN) 9. the European Society for Clinical Nutrition and Metabolism (ESPEN). |
