# Supplementary material for: A systematic scoping review on the consequences of stress-related hyperglycaemia
Source: PLoS One. 2018 Apr 6;13(4):e0194952. doi: 10.1371/journal.pone.0194952 (PMC5889160; doi:10.1371/journal.pone.0194952)
Supplement: S2 Table — aRespiratory, genito-urinary, bloodstream; brespiratory infections; cgenitourinary tract infections; dblood infections; eintra-abdominal infections; fskin/soft tissue infections; grespiratory infections, genito-urinary tract infections, wound or skin infections; hpneumonia, urinary tract infections, bacteraemia, intra-abdominal abscess, wound infection, open fracture infection; ipneumonia, line sepsis, bacteraemia, wound infection, abscess; joverall ICU mortality; kweek 1 mortality; lweek 2 mortality; mweek 3 mortality; nbacteraemia; ourinary tract infections; ppneumonia in the third week; qsurgical site infections; rpneumonia, urinary tract infection, bloodstream infection, surgical site infection, intra-abdominal abscess, Clostridium difficile colitis, meningitis, and sinusitis; sskin/wound infections; tother infections; upneumonia; vwound infections. BG, blood glucose; CI, confidence interval; CIT, continuous intravenous regular human insulin infusion; HG, hyperglycaemia; HGI, hyperglycaemic index; HR, hazard ratio; ICU = intensive care unit; IIT, intensive insulin control; IQR = interquartile range; ISS = injury severity score; max, maximum; NA = not applicable; NPH = neutral protamine Hagedorn; NR, not reported; OR, odds ratio; ORa, odds ratio adjusted; PH, persistent hyperglycaemia; RR, risk ratio; RRa, risk ratio adjusted; SD, standard deviation; SE, standard error; SEM, standard error of the mean; SIT, supplemental intermittent intravenous regular human insulin therapy; TBI, traumatic brain injury; TIR-hi, time in targeted blood glucose range above the median value; TIR-lo, time in targeted blood glucose range below the median value. *P<0.05; **P<0.01; †Nosocomial infections. (DOC) [file pone.0194952.s004.doc]

**Supplementary Table 2. Mortality, infections, length of stay, and resource use in patients with or without hyperglycaemia in trauma and mixed ICUs**

| Study | Sample size | Method BG | BG threshold | Patients with/ without HG (n) | Mortality n/N (%) | OR/HR for mortality (95% CI) | Infections  n/N (%) | ICU length of stay, days (mean,SD) | Hospital length of stay, days | Time on mechanical ventilation, days | Blood transfusions | Renal replacement therapy, n (%) |
| --- | --- | --- | --- | --- | --- | --- | --- | --- | --- | --- | --- | --- |
| Trauma ICU |  |  |  |  |  |  |  |  |  |  |  |  |
| Bahloul et al., 2004^[64]^ | 437 | NR | 180 mg/dL | Patients with BG >180 mg/dL (144) | 71/144 (49.3) | OR=3.28 (1.84–5.84) | NR | NR | NR | NR | NR | NR |
|  |  |  |  | Patients with BG <180 mg/dL (293) | NR |  | NR | NR | NR | NR | NR | NR |
| Bochicchio et al., 2005^[38]^ | 252 | NR | 200 mg/dL | Patients with BG >200 mg/dL (54) | 10/54 (19) | NR | 26/54 (48.0)*^a^ | 9.90 (mean) NR (NR)* | 18.70 days (mean)  NR (NR)* | NR | First 24 hours:  3.7 (mean)  2.5 (SD) | NR |
|  |  |  |  | Patients with BG <200 mg/dL (198) | 18/198 (9) |  | 57/198 (29.0)^a^ | 6.60 (mean)  NR (NR) | 15.10 (mean)  NR (NR) | NR | First 24 hours:  3.1 (mean)  2.3 (SD) | NR |
| Bochicchio et al., 2005^[39]^ | 942 | NR | NR | Patients with all high pattern of glucose control (26) | NR (31.5) | OR=1.4 (1.4–10.0)** | NR (50.0)^b^ NR (31.0)^c^ NR (23.0)^d^ NR (23.0)^e^ NR (3.8)^f^ | 14.00 (mean)  8.00 (SD)* | 22.00 (mean)  13.00 (SD)* | 16.00 (mean)  12.00 (SD)* | NR | NR |
|  |  |  |  | Patients with all moderate pattern of glucose control (39) | NR (20.5) | OR=1.0 (1.0–6.2)* | NR (46.0)^b^ NR (26.0)^c^ NR (26.0)^d^ NR (3.0)^e^ NR (7.7)^f^ | 15.00 (mean)  8.00 (SD)* | 21.00 (mean)  9.00 (SD)* | 14.00 (mean)  9.00 (SD)* | NR | NR |
|  |  |  |  | Patients with all low pattern of glucose control (306) | NR (6.8) | NA | NR (27.0)^b^ NR (16.0)^c^ NR (12.0)^d^ NR (6.2)^e^ NR (7.5)^f^ | 10.00 (mean)  7.00 (SD) | 15.00 (mean)  10.00 (SD) | 8.70 (mean)  7.00 (SD) | NR | NR |
| Bochicchio et al., 2007^[42]^ | 896 | Point of care testing; routine laboratory analysis; highest serum BG recorded daily | >140 mg/dL; 220 mg/dL | Patients with BG >220 mg/dL (14) | 6/14 (43) | Week 1: OR=17.1 (4.0–73.3) Week 2: OR=3.7 (0.6–21.8) Week 3: OR=4.9 (0.5–53.0) | 6/14 (43.0)^g^ | 15.00 (mean)  14.00 (SD)* | 21.00 (mean)  27.00 (SD)* | 18.00 (mean)  12.00 (SD)* | NR | NR |
|  |  |  |  | Patients with BG 140–219 mg/dL (157) | 29/157 (18) | Week 1: OR=4.5 (1.6–12.4);  Week 2: OR=3.3 (1.2–9.5);  Week 3: OR=12.2 (3.8–39.0); | 96/157 (61.0)^g^ | 17.00 (mean) 11.00 (SD)* | 23.00 (mean)  12.00 (SD)* | 17.00 (mean)  12.00 (SD)* | NR | NR |
|  |  |  |  | Patients with BG 0–139 mg/dL (226) | 6/226 (2) | NA | 84/226 (37.0)^g^ | 10.00 (mean) 8.00 (SD) | 16.00 (mean)  11.00 (SD) | 9.00 (mean)  8.00 (SD) | NR | NR |
| Chabok et al., 2009^[65]^ | 115 | NR | ≥200 mg/dL | Patients with traumatic head injury and BG ≥200 mg/dL (42) | 27 (64.3) | NR | NR | 11.30 (mean) 14.80 (SD) | 17.95 (mean)  20.71 (SD) | NR | NR | NR |
|  |  |  |  | Patients with traumatic head injury and BG <200 mg/dL (73) | 31 (42.5) |  | NR | 10.45 (mean) 10.87(SD) | 22.32 (mean)  23.30 (SD) | NR | NR | NR |
| Diaz et al., 2009^[47]^ | 1,334 | NR | ≥150 mg/dL | Patients with HG (345) | 30/345 (8.7) | ORa=1.97 (SE=0.54) | NR | 9.00 (median)  6–16 (25–75 percentile) | NR | NR | NR | NR |
|  |  |  |  | Patients without HG (989) | 33/989 (3.3) |  | NR | 9.00 (median)  5–16 (25–75 percentile) | NR | NR | NR | NR |
| Dickerson et al., 2013^[55]^ | 66 | NR | >150 mg/dL; 170 mg/dL; 180 mg/dL | Hyperglycaemic patients with trauma injury (CIT + NPH) (32) | 1/32 (3.1) | NR | NR | 34.00 (mean) 21.00 (SD) | 41.00 (mean)  26.00 (SD) | 25.00 (mean)  19.00 (SD) | NR | NR |
|  |  |  |  | Hyperglycaemic patients with trauma injury (SIT + NPH) (34) | 4/34 (11.7) | NR | NR | 30.00 (mean) 15.00 (SD) | 39.00 (mean)  28.00 (SD) | 21.00 (mean)  15.00 (SD) | NR | NR |
| Duane et al., 2006^[41]^ | 226 | NR | >150mg/dL | Trauma patients with BG >150mg/dL (50) | NR (30) | NR | NR (12.5)^h^ | 1.90 (NR)  3.00 (NR)* | 4.90 (mean)  5.50 (SD)* | 1.00 (mean)  2.30 (SD)* | NR | NR |
|  |  |  |  | Trauma patients with BG <150mg/dL (176) | NR (5.7) |  | NR (5.7)^h^ | 1.00 (NR) 2.80 (NR) | 3.50 (mean)  4.40 (SD) | 0.10 (mean)  0.60 (SD) | NR | NR |
| Duane et al., 2008^[45]^ | 335 | NR | >150 mg/dL | Patients with high BG >150 mg/dL (68) | 10/68 (14.7) | NR | NR | 13.10 (NR)  14.90 (NR) | 13.10 (mean)  14.90 (SD) | 1.00 (mean)  3.10 (SD) | NR | NR |
|  |  |  |  | Patients with low BG ≤150 mg/dL (267) | 26/267 (9.7) |  | NR | 5.70 (NR)  7.90 (NR) | 11.40 (mean)  12.20 (SD) | 1.30 (mean)  3.50 (SD) | NR | NR |
| Gale et al., 2007^[44]^ | 103 | Laboratory serum glucose; point-of-care bedside finger stick | NR | Injured patients with average BG >140 mg/dL (27) | NR (22.2) | NR | NR | 10.00 (mean) 9.00 (SD) | 20.00 (mean)  20.00 (SD) | 8.00 (mean)  7.00 (SD) | NR | NR |
|  |  |  |  | Injured patients with average BG ≤140 mg/dL (76) | NR (9.1) |  | NR | 9.00 (mean)  11.00 (SD) | 17.00 (mean)  13.00 (SD) | 10.00 (mean)  13.00 (SD) | NR | NR |
|  |  |  |  | Non-diabetic injured patients with average BG >140 mg/dL (19) | NR (31.6) |  | NR | 10.00 (mean)  9.00 (SD) | 20.00 (mean)  22.00 (SD) | 9.00 (mean)  7.00 (SD) | NR | NR |
|  |  |  |  | Non-diabetic injured patients with average BG ≤140 mg/dL (71) | NR (9.9) |  | NR | 10.00 (mean)  11.00 (SD) | 17.00 (mean)  13.00 (SD) | 11.00 (mean)  14.00 (SD) | NR | NR |
| Jeremitsky et al., 2003^[33]^ | 81 | NR | >200 mg/dL | Hyperglycaemic blunt trauma patients (32) | NR (51.6) | NR | NR | NR | NR | NR | NR | NR |
|  |  |  |  | Normoglycaemic blunt trauma patients (49) | NR (28) |  | NR | NR | NR | NR | NR | NR |
| Kreutziger et al., 2015^[59]^ | 279 | Laboratory or blood gas testing within 20 min after admission | >169 mg/dL | Trauma patients with an ISS ≥17 and admission BG >169 mg/dL (75) | 20 (26.7) | NR | NR | NR | NR | NR | NR | NR |
|  |  |  |  | Trauma patients with an ISS ≥17 and admission BG <169 mg/dL (204) | 22 (10.8) |  | NR | NR | NR | NR | NR | NR |
| Laird et al., 2004^[36]^ | 516 | NR | >110 mg/d;>150 mg/d; >200 mg/dL | Patients with early BG measurements of ≥110 mg/dL (483) | 82/483 (17) | NR | NR | NR | NR | NR | NR | NR |
|  |  |  |  | Patients with early BG measurements of <110 mg/dL (33) | 3/33 (9) |  | NR | NR | NR | NR | NR | NR |
|  |  |  |  | Patients with early BG measurements of ≥150 mg/dL (311) | 70/311 (23) |  | NR | NR | NR | NR | NR | NR |
|  |  |  |  | Patients with early BG measurements of <150 mg/dL (205) | 15/205 (7) |  | NR | NR | NR | NR | NR | NR |
|  |  |  |  | Patients with early BG measurements of ≥200 mg/dL (90) | 31/90 (34) |  | 29/90 (32.0)*^i^ | NR | NR | NR | NR | NR |
|  |  |  |  | Patients with early BG measurements of <200 mg/dL (426) | 54/426 (13) |  | 96/426 (22.0)^i^ | NR | NR | NR | NR | NR |
| Lionel et al., 2014^[67]^ | 120 | Point of care glucometers | ≥200 mg/dL | All trauma patients with admission BG ≥200 mg/dL (26) | 4/26 (15.4) | NR | NR | NR | NR | NR | NR | NR |
|  |  |  |  | All trauma patients with admission BG <200 mg/dL (87) | 12/87 (13.8) |  | NR | NR | NR | NR | NR | NR |
| Meier et al., 2008^[60]^ | 228 | Blood gas analyser; BG measured every 4 hours or at shorter intervals | >180 mg/dL | Patients with severe TBI whose BG levels were maintained between 63 mg/dL and 117 mg/dL (114) | 29/114 (25)^j^ | NR | NR (25.0)*^n^ NR (22.0)*^o^ | Survivors: 17.00 (median)  2.00–48.00 (range);  deceased: 9.00 (median)  2.00–23.00 (range) | NR | NR | NR | NR |
|  |  |  |  | Patients with severe TBI whose BG levels were maintained between 63 mg/dL and 117 mg/dL (114) | 12/114 (11)^k^ |  | NR | NR | NR | NR | NR | NR |
|  |  |  |  | Patients with severe TBI whose BG levels were maintained between 63 mg/dL and 117 mg/dL (89) | 9/89 (10)^l^ |  | NR (18.0)*^p^ | NR | NR | NR | NR | NR |
|  |  |  |  | Patients with severe TBI whose BG levels were maintained between 63 mg/dL and 117 mg/dL (57) | 8/57 (14)^m^ |  | NR | NR | NR | NR | NR | NR |
|  |  |  |  | Patients with severe TBI whose BG levels were maintained between 90 mg/dL  and 144 mg/dL (114) | 22/114 (19)^j^ |  | NR (18.0)^n^ NR (16.0)^o^ | Survivors: 15.00 (median)  2.00–52.00 (range); deceased: 11.00 (median)  2.00–43.00 (range) | NR | NR | NR | NR |
|  |  |  |  | Patients with severe TBI whose BG levels were maintained between 90 NRmg/dL and 144 mg/dL (114) | 8/114 (7)^k^ |  | NR | NR | NR | NR | NR | NR |
|  |  |  |  | Patients with severe TBI whose BG levels were maintained between 90 mg/dL and 144 mg/dL (86) | 5/86 (6)^l^ |  | NR (26.0)^p^ | NR | NR | NR | NR | NR |
|  |  |  |  | Patients with severe TBI whose BG levels were maintained between 90 mg/dL and 144 mg/dL (57) | 9/57 (16)^m^ |  | NR | NR | NR | NR | NR | NR |
| Richards et al., 2013^[57]^ | 187 | Fingerstick, serum levels of glucose | ≥200 mg/dL | Trauma patients with isolated orthopaedic injuries requiring acute operative intervention and HG (more than 1 random BG ≥200 mg/dL) (33) | NR | NR | 1 (3.0)^q^ | NR | NR | NR | NR | NR |
|  |  |  |  | Trauma patients with isolated orthopaedic injuries requiring acute operative intervention and BG <200 mg/dL (154) | NR |  | 15 (9.7)^q^ | NR | NR | NR | NR | NR |
|  |  |  |  | Trauma patients with HGI >1.72 (41) | NR |  | 8 (19.5)^q^ | NR | NR | NR | NR | NR |
|  |  |  |  | Trauma patients with HGI ≤1.72 (146) | NR |  | 8 (5.5)^q^ | NR | NR | NR | NR | NR |
| Safavi et al., 2009^[66]^ | 600 | NR | >198 mg/dL | Patients ≥16 years, ≥3 ICU days, BG >198 mg/dL (NR) | NR | NR | NR | 6.10 (mean) 0.54 (SEM) | NR | NR | NR | NR |
|  |  |  |  | Patients ≥16 years, ≥3 ICU days, BG <198 mg/dL (NR) | NR |  | NR | 6.10 (mean) 0.37 (SEM) | NR | NR | NR | NR |
| Salim et al., 2009^[48]^ | 834 | Laboratory testing | ≥150 mg/dL | Blunt trauma patients with PH (105) | 67/105 (64) | OR crude=6.81 (4.40–10.53) ORa=4.91 (2.88–8.56) | NR | NR | NR | NR | NR | NR |
|  |  |  |  | Blunt trauma patients without PH (729) | 150/729 (21) | NR | NR | NR | NR | NR | NR | NR |
| Santhanam et al., 2007^[68]^ | 208 | NR | >100 mg/dL | Head injury patients with BG >110 mg/dL (30) | 14/30 (47) | NR | NR | NR | NR | NR | NR | NR |
|  |  |  |  | Head injury patients without HG (178) | 50/178 (28) |  | NR | NR | NR | NR | NR | NR |
| Shin et al., 2007^[43]^ | 609 | Measured hourly | ≥150 mg/dL | Trauma patients with admission BG ≥150 mg/dL (290) | NR (18.5) | NR | NR (43.2)*^r^ | 9.70 (NR)  NR (NR) | 18.90 (NR)  NR (NR) | 5.10 (mean) | NR | NR |
|  |  |  |  | Trauma patients with admission BG <150 mg/dL (319) | NR (8.2) |  | NR (27.6)^r^ | 8.10 (NR)  NR (NR) | 17.30 (NR)  NR (NR) | 4.40 (mean) | NR | NR |
| Smith et al., 2012^[53]^ | 30 | During first 72 hours after admission BG measured hourly | >150 mg/dL | Trauma patients with HG (19) | 7/19 (36.8) | NR | NR | 12.05 (mean) 17.56 (SD) | 30.32 (mean)  43.82 (SD) | 9.21 (mean)  10.51 (SD) | NR | NR |
|  |  |  |  | Trauma patients without HG (11) | 0/11 (0) |  | NR | 9.27 (mean)  9.23 (SD) | 15.64 (mean) 12.82 (SD) | 5.82 (mean) 10.33 (SD) | NR | NR |
| Sperry et al., 2009^[49]^ | 862 | NR | ≥130 mg/dL | Trauma patients with PH (401) | NR (16.8) | Group I (130–145 mg/dL) HR=0.95 (0.54–1.67) Group II (146–165 mg/dL) HR=1.88 (1.02–3.46) Group III (>165 mg/dL) HR=1.99 (1.12–3.51) | NR | 15.70 (mean)  13.00 (SD)* | 24.50 (mean)  19.00 (SD) | 12.30 (mean)  11.00 (SD)* | Blood transfusion (>6 units): 48.5% | NR |
|  |  |  |  | Trauma patients without PH (461) | NR (9.7) |  | NR | 13.60 (mean) 13.00 (SD) | 23.90 (mean)  19.00 (SD) | 10.20 (mean)  12.00 (SD) | Blood transfusion (>6 units): 46.3% | NR |
| Sung et al., 2005^[37]^ | 1,003 | BG measured on admission | >200mg/dL | Critically ill trauma patients with BG >200 mg/dL (255) | 66/255 (26) | RR=2.2 (1.4–3.4) | 49/255 (19.0)*^b^ 29/255 (11.0)*^c^ 25/255 (10.0)*^d^ 10/255 (4.0)^e^ 16/255 (6.0)^s^ 3/255 (1.0)^t^ | 13.40 (mean)  11.00 (SD) | 17.80 (mean)  15.00 (SD)* | 13.00 (mean) 12.00 (SD)* | NR | NR |
|  |  |  |  | Critically ill trauma patients with BG <200 mg/dL (748) | 92/748 (12) |  | 81/748 (11.0)^b^ 53/748 (7.0)^c^  42/748 (6.0)^d^ 31/748 (4.0)^e^ 25/748 (3.0)^s^ 8/748 (1.0)^t^ | 12.00 (mean)  10.00 (SD) | 14.70 (mean)  12.00 (SD) | 11.00 (mean)  11.00 (SD) | NR | NR |
| Yendamuri et al., 2003^[34]^ | 738 | NR | >200 mg/dL | Trauma patients with BG >200 mg/dL (41) | NR (34.1) | OR=4.76 (1.55–14.20) | 6/41 (14.6)*^u^ 3/41 (7.3)^o^ 2/41 (4.9)*^v^ 3/41 (7.3)*^n^ | 6.76 (mean)  NR (NR)* | 14.88 (mean)  NR (NR) | NR | NR | NR |
|  |  |  |  | Trauma patients with BG <200 mg/dL (697) | NR (3.7) |  | 22/697 (3.2)^u^ 17/697 (2.4)^o^ 4/697 (0.6)^v^ 12/697 (1.7)^n^ | 1.55 (mean)  NR (NR) | 5.40 (mean)  NR (NR) | NR | NR | NR |
|  |  |  | >135 mg/dL | Trauma patients with BG >135 mg/dL (181) | NR (15.5) | OR=3.61 (1.35–10.00) | 17/181 (9.4)*^u^ 12/181 (6.6%)*^o^ 2/181 (1.1)*^v^ 9/181 (5.0)*^n^ | NR | NR | NR | NR | NR |
|  |  |  | NA | Trauma patients with BG <135 mg/dL (557) | NR (2) |  | 11/557 (2.0)^u^ 8/557 (1.4)^o^ 4/557 (0.7)^v^ 6/557 (1.1)^n^ | NR | NR | NR | NR | NR |
| Mixed ICU |  |  |  |  |  |  |  |  |  |  |  | NR |
| Van Ackerbroeck et al., 2015^[61]^ | 338 | Arterial line; on-site blood gas analyser | >140 mg/dL | Medical/surgical ICU patients with stress HG (246) | NR | NR | NR | 99 hours (median)  37–503 (range)* | NR | NR | NR | NR |
|  |  |  |  | Medical/surgical ICU patients without stress HG (92) | NR |  | NR | 82 hours (median) 39–1,075 (range) | NR | NR | NR | NR |
| Krinsley and Preiser, 2015^[58]^ | 3,297 | ACCU-CHEK Inform II glucose meters; BG capillary, venous, or arterial blood measured every 3 hours | ≥180 mg/dL | Non-diabetic TIR-hi patients with max. BG ≥180 mg/dL (234) | NR (15.8) | NR | NR | NR | NR | NR | NR | NR |
|  |  |  |  | Non-diabetic TIR-lo patients with max. BG ≥180 mg/dL (801) | NR (18.7) |  | NR | NR | NR | NR | NR | NR |
|  |  |  |  | Diabetic TIR-hi patients with max. BG ≥180 mg/dL (279) | NR (16.5) |  | NR | NR | NR | NR | NR | NR |
|  |  |  |  | Diabetic TIR-lo patients with max. BG ≥180 mg/dL (346) | NR (14.5) |  | NR | NR | NR | NR | NR | NR |
| Lucas et al., 2012^[69]^ | 386 | BG measured four times per day; glucometer | >160 mg/dL | Patients with HG (52) | 16/52 (30.8) | NR | NR | NR | NR | NR | NR | NR |
|  |  |  |  | Patients without HG (334) | 72/334 (22.6) |  | NR | NR | NR | NR | NR | NR |
| Leite et al., 2010^[70]^ | 779 | Plasma BG measured with enzymatic colouri­metric method; capillary BG measure­ments | >110 mg/dL;  >180 mg/dL | Patients with abnormal BG (149) | 24/149 (16.1) | NR | NR | NR | NR | NR | NR | NR |
|  |  |  |  | Patients with normal BG (71) | 2/71 (2.8) |  | NR | NR | NR | NR | NR | NR |
| Donati et al., 2014^[62]^ | 2,782 | Near-patient capillary tests (every 2 hours); arterial blood gas analyses (at least every 8 hours); central laboratory determi­na­tions (at least once per day) | >150 mg/dL | Patients with >3 BG measurements and insulin treatment (1,143) | 258/1,143 (22.6) | NR | 518/1,143 (45.3) | 8.7 days (median)  3.7–17.5 (IQR)* | NR | NR | NR | NR |
|  |  |  |  | Patients with >3 BG measurements and no insulin treatment (1,639) | 223/1,639 (13.6) |  | 347/1,639 (21.2) | 3.8 days (median)  1.8–7.9 (IQR) | NR | NR | NR | NR |
| Krinsley et al., 2013^[56]^ | 44,964 | BG monitor: arterial blood gas analyser, ACCU-CHEK Inform glucose meters and/or central laboratory analyser; source of blood: arterial, venous, and/or capillary | NR | Non-diabetic patients with mean BG ≥180 mg/dL (1,846) | NR (23.5) | Medical patients: mean BG 80–140 mg/dL range indepen­dently associ­ated with the lowest risk of mortality; diabetic patients: mean BG 110–140 mg/dL, 140–180 mg/dL, and <180 mg/dL had a reduced risk of mortality | NR | NR | NR | NR | NR | NR |
|  |  |  |  | Non-diabetic patients with mean BG 140–180 mg/dL (9,003) | NR (14.2) |  | NR | NR | NR | NR | NR | NR |
|  |  |  |  | Non-diabetic patients with mean BG 110 - 140 mg/dL (15256) | NR (11.1) |  | NR | NR | NR | NR | NR | NR |
|  |  |  |  | Non-diabetic patients with mean BG 80–110 mg/dL (5,749) | NR (10.5) |  | NR | NR | NR | NR | NR | NR |
|  |  |  |  | Diabetic patients with mean BG ≥180 mg/dL (3,389) | NR (14.0) |  | NR | NR | NR | NR | NR | NR |
|  |  |  |  | Diabetic patients with mean BG 140–180 mg/dL (4,827) | NR (12.6) |  | NR | NR | NR | NR | NR | NR |
|  |  |  |  | Diabetic patients with mean BG 110–140 mg/dL (3,555) | NR (12.6) |  | NR | NR | NR | NR | NR | NR |
|  |  |  |  | Diabetic patients with mean BG 80–110 mg/dL (1,035) | NR (15.3) |  | NR | NR | NR | NR | NR | NR |
| Badawi et al., 2012^[54]^ | 194,772 | Laboratory interfaces and point-of-care testing | >180 mg/dL; 150 mg/dL | Patients with ICU-acquired HG (31,375) | 3,108/ 31,375 (9.9) | RRa=1.13 (1.04–1.58) | NR | 5.7 days (median)  3.5–9.9 (IQR) | 12.0 (median)  7.5–19.5 (IQR) | 0.7 (median) 0.0–5.5 (IQR) | NR | NR |
|  |  |  |  | Patients without ICU-acquired HG (70,502) | 2,091/ 70,502 (3.0) |  | NR | 3.1 days (median)  2.5–4.7 (IQR) | 7.6 days (median)  4.9–12.1 (IQR) | 0.0 (median) 0.0-0.6 (IQR) | NR | NR |
| Siegelaar et al., 2010^[3]^ | 5,828 | Arterial blood samples, hand-held glucose measuring device | ≥153 mg/dL;  ≥171 mg/dL | Medical ICU patients with BG ≤119 mg/dL (268) | NR (26.9) | ICU admission BG 128–135 mg/dL (mean) OR=1.00 | NR | NR | NR | NR | NR | NR |
|  |  |  |  | Medical ICU patients with BG in the safe range 121–153 mg/dL (804) | NR (14.1) | ICU admission 137–151 mg/dL (mean): OR=1.30 (0.70–2.20) | NR | NR | NR | NR | NR | NR |
|  |  |  |  | Medical ICU patients with BG ≥153 mg/dL (267) | NR (35.6) | ICU admission ≥153 mg/dL (mean): OR=3.00 (1.80–5.10) | NR | NR | NR | NR | NR | NR |
|  |  |  |  | Surgical ICU patients with BG ≤119 mg/dL (898) | NR (3.6) | ICU admission 126–133 mg/dL (mean): OR=3.30 (0.70–15.60) | NR | NR | NR | NR | NR | NR |
|  |  |  |  | Surgical ICU patients with BG in the safe range 126–169 mg/dL (2,694) | NR (1.0) | ICU admission 135–148 mg/dL (mean): OR=3.20 (0.70–15.50) | NR | NR | NR | NR | NR | NR |
|  |  |  |  | Surgical ICU patients with BG ≥171 mg/dL (897) | NR (1.4) | ICU admission 149–169 mg/dL (mean): OR=1.00 | NR | NR | NR | NR | NR | NR |
|  |  |  |  |  |  | ICU admission ≥171 mg/dL (mean): OR=6.20 (1.40–28.30) | NR | NR | NR | NR | NR | NR |
| Juneja et al., 2009^[51]^ | 4,588 | Hourly BG measure­ments; fingerstick capillary sampling; venous and arterial sampling with point-or-care glucometer, blood gas analyser, central laboratory measure­ment | NR | HG ICU patients treated with GlucoStabilizer to BG target 79–110 mg/dL (4,588) | NR | NR | NR | 5.5 days (NR) NR (NR) | NR | NR | NR | NR |
| Falciglia et al., 2009^[50]^ | 259,040 | Multi-sample biochem­istry laboratory panel | 110 mg/dL; 145 mg/dL;  200 mg/dL;  300 mg/dL | Patients with mean BG 70–110 mg/dL (77,376) | NR (7.3) | NR | NR | NR | NR | NR | NR | NR |
|  |  |  |  | Patients with mean BG 111–145 mg/dL (96,399) | NR (10.2) | OR=1.31 (1.26–1.36) | NR | NR | NR | NR | NR | NR |
|  |  |  |  | Patients with mean BG levels 146–199 mg/dL (57,437) | NR (14.8) | OR=1.82 (1.74–1.90) | NR | NR | NR | NR | NR | NR |
|  |  |  |  | Patients with mean BG 200–300 mg/dL (23,821) | NR (17.3) | OR=2.13 (2.03–2.25) | NR | NR | NR | NR | NR | NR |
|  |  |  |  | Patients with mean BG >300 mg/dL (4,007) | NR (21.9) | OR=2.85 (2.58–3.14) | NR | NR | NR | NR | NR | NR |
| Jacka et al., 2009^[52]^ | 606 | BG at admission and daily; finger capillary sampling; IIT patients: BG measured every 6 hours, and hourly until stable | ≥180 mg/dL | Critically ill hyperglycaemic patients with a primary neurological diagnosis (58) | 16/58 (21.3) | Any episode of HG: crude OR=1.51 (0.78–2.92); ORa=1.05 (0.41–2.72); no. of episodes (per episode): crude OR=1.05 (1.01–1.09); ORa=1.04 (1.00–1.09) | NR | 4.0 days (median)  1.0–12.0 (IQR)* | 13.0 (median 8.0–34.0 (IQR)* | NR | NR | NR |
|  |  |  |  | Critically ill normoglycaemic patients with a primary neurological diagnosis (543) | 87/543 (16.0) | NR | NR | 2 days (median) 1–4 (IQR) | 8.0 days (median)  3.0–18.0 (IQR) | NR | NR | NR |
| Lacherade et al., 2007^[63]^ | 105 | Capillary BG level; ACCU-CHEK Inform; measured every 1–3 hours | >126 mg/dL | Patients with mean BG >126 mg/dL (105) | ICU mortality: NR (33.3);  hospital mortality: NR (41.9) | NR | 23/105 (22.0) ^†^ | 10.0 days (median)  6.0–18.0 (IQR) | NR | 8.0 (median)  5.0–17.0 (IQR) | NR | 4 (NR) |
| Whitcomb et al., 2005^[40]^ | 2,713 | BG measured on admission | >200 mg/dL | Hyperglycaemic patients (743) | 77/743 (10.4) | OR=1.42 (95% CI: 1.09, 1.86) | NR | NR | 12.2 (mean) 13.3 (SD) | NR | NR | NR |
|  |  |  |  | Normoglycaemic patients (1,970) | 157/1,970 (8.0) | NR | NR | 10.5 days (mean)  11.7 (SD) | NR | NR | NR | NR |
| Krinsley et al., 2003^[35]^ | 1,826 | NR | NR | Critically ill patients with mean BG 80–99 mg/dL (264) | NR (9.6) | NR | NR | NR | NR | NR | NR | NR |
|  |  |  |  | Critically ill patients with mean BG 100–119 mg/dL (491) | NR (12.2) | NR | NR | NR | NR | NR | NR | NR |
|  |  |  |  | Critically ill patients with mean BG 120–139 mg/dL (338) | NR (15.1) | NR | NR | NR | NR | NR | NR | NR |
|  |  |  |  | Critically ill patients with mean BG 140–159 mg/dL (202) | NR (18.8) | NR | NR | NR | NR | NR | NR | NR |
|  |  |  |  | Critically ill patients with mean BG 160–179 mg/dL (141) | NR (28.4) | NR | NR | NR | NR | NR | NR | NR |
|  |  |  |  | Critically ill patients with mean BG 180–199 mg/dL (102) | NR (29.4) | NR | NR | NR | NR | NR | NR | NR |
|  |  |  |  | Critically ill patients with mean BG 200–249 mg/dL (144) | NR (37.5) | NR | NR | NR | NR | NR | NR | NR |
|  |  |  |  | Critically ill patients with mean BG 250–299 mg/dL (70) | NR (32.9) | NR | NR | NR | NR | NR | NR | NR |
|  |  |  |  | Critically ill patients with mean BG >300 mg/dL (40) | NR (42.5) | NR | NR | NR | NR | NR | NR | NR |
| Umpierrez et al., 2002^[32]^ | 1,886 | NR | >126 mg/dL;  200 mg/dL | Patients with new HG (223) | 20/223 (8.9) | 18.3-fold increased mortality rate | NR | NR | NR | NR | NR | NR |
|  |  |  |  | Patients with known diabetes (495) | 8/495 (1.6) | 2.7-fold increased mortality rate | NR | NR | NR | NR | NR | NR |
|  |  |  |  | Patients with normoglycaemia (1,168) | 11/1,168 (0.9) | NR | NR | NR | NR | NR | NR | NR |
| Levin et al., 2008^[46]^ | 170 | Laboratory BG measures | >150 mg/dL | Patients with BG >150 mg/dL (136) | 38/136 (28.0) | NR | NR | 15.0 days (median)  NR (NR)* | NR | NR | 0.4 (mean) 1.9 (SD) per hyper-glycaemic day* | NR |
|  |  |  |  | Patients with BG ≤150 mg/dL (34) | 6/34 (18.0) | NR | NR | 10 days (median)  NR (NR) | NR | NR | 0.2 (mean)  0.6 (SD) per non-hyper-glycaemic day | NR |
| Plummer et al., 2014^[71]^ | 1,000 | Blood gas analysers; bedside glucometer; BG measured hourly | >180 mg/dL | Patients with critical illness-associated HG (498) | ICU mortality: 73/498 (15.0);  hospital mortality: 84/498 (17.0) | NR | NR | 3.0 days (median) 1.8–7.9 (IQR)* | 13.9 (median)  7.2–31.0 (IQR)* | NR | NR | NR |
|  |  |  |  | Normoglycaemic patients (227) | ICU mortality: 15/227 (7.0);  hospital mortality: 17/227(7.0) | NR | NR | 2 days (median)  1.3–4.4 (IQR) | 11.3 days (median)  5.6–23.3 (IQR) | NR | NR | NR |
| Schlussel et al., 2011^[30]^ | 395 | Point-of-care testing: hourly BG capillary blood samples; daily serum chemistries and arterial blood gases (venous, arterial blood samples) | ≥141 mg/dL | Patients with uncontrolled BG (153) | 30/153 (20.0) | NR | NR | 10.0 days (mean)  0.8 (SD) | 24.0 (mean)  2.3 (SD) | NR | NR | NR |
|  |  |  |  | Patients with controlled BG (82) | 17/82 (21.0) | NR | NR | 10 days (mean)  1.2 (SD) | 22.0 days (mean)  2.5 (SD) | NR | NR | NR |
